# Administration of β-lactam antibiotics and delivery method correlate with intestinal abundances of *Bifidobacteria* and *Bacteroides* in early infancy, in Japan

**DOI:** 10.1038/s41598-021-85670-z

**Published:** 2021-03-18

**Authors:** Naruaki Imoto, Chie Kano, Yumi Aoyagi, Hiroto Morita, Fumitaka Amanuma, Hidekazu Maruyama, Shuko Nojiri, Naoyuki Hashiguchi, Shin Watanabe

**Affiliations:** 1 Department of Microbiome Research, School of Medical Science, Juntendo University, Hongou 2-1-1, Bunkyo Ward, Tokyo 113-8421 Japan; 2 Core Technology Laboratories, Asahi Group Holdings, Ltd., Sagamihara, Kanagawa Japan; 3 Department of Paediatrics, Department of Neonatology, Iwate Prefectural Iwai Hospital, Ichinoseki, Iwate Japan; 4 Juntendo Clinical Research Support Centre, Juntendo University, Bunkyo Ward, Tokyo Japan; 5 Department of Emergency and Disaster Medicine, School of Medical Science, Juntendo University, Bunkyo Ward, Tokyo Japan

**Keywords:** Microbiome, Antibiotics, Microbiota

## Abstract

The intestinal microbiome changes dynamically in early infancy. Colonisation by *Bifidobacterium* and *Bacteroides* and development of intestinal immunity is interconnected. We performed a prospective observational cohort study to determine the influence of antibiotics taken by the mother immediately before delivery on the intestinal microbiome of 130 healthy Japanese infants. Faecal samples (383) were collected at 1, 3, and 6 months and analysed using next-generation sequencing. Cefazolin was administered before caesarean sections, whereas ampicillin was administered in cases with premature rupture of the membranes and in Group B *Streptococcus*-positive cases. *Bifidobacterium* and *Bacteroides* were dominant (60–70% mean combined occupancy) at all ages. A low abundance of *Bifidobacterium* was observed in infants exposed to antibiotics at delivery and at 1 and 3 months, with no difference between delivery methods. A lower abundance of *Bacteroides* was observed after caesarean section than vaginal delivery, irrespective of antibiotic exposure. Additionally, occupancy by *Bifidobacterium* at 1 and 3 months and by *Bacteroides* at 3 months differed between infants with and without siblings. All these differences disappeared at 6 months. Infants exposed to intrapartum antibiotics displayed altered *Bifidobacterium* abundance, whereas abundance of *Bacteroides* was largely associated with the delivery method. Existence of siblings also significantly influenced the microbiota composition of infants.

## Introduction

The human body is inhabited by 100–1000 trillion bacteria in the oral cavity, skin, and intestine, influencing the biological health of the host^1, 2^. There are large differences in the composition of the human intestinal microbiome depending on race, country, and lifestyle^3–5^. Although the predominance of *Bifidobacterium* in the intestinal microbiome has been identified in Japanese children, there are very few studies on the intestinal microbiome specifically in Japanese infants^5–10^.

Many studies have indicated an association between the intestinal microbiome and disease^11–13^. Colonisation by intestinal bacteria in early infancy is known to have a major effect on intestinal immunity^14–16^. In particular, colonisation by the dominant bacteria such as *Bifidobacteria* and *Bacteroides*^17–23^, resulting from the start of food ingestion at 6 months after birth has been implied to be linked with the onset of diseases such as allergies^24–26^. Various factors are known to influence bacterial colonisation, including maternal exposure to antibiotics administered as intrapartum antimicrobial prophylaxis (IAP) in Group B *Streptococcus* (GBS)-positive mothers^27–29^ and antibiotic administration in the late perinatal period^30^. These studies, however, have reported differences in the administration and screening period, and offer inconclusive results. The influence of the delivery method on the *Bifidobacteria* and *Bacteroides* populations in infants has also been extensively studied^16, 31–33^. It is a widely accepted hypothesis that as infants delivered by caesarean section do not pass through the birth canal, they do not come in contact with the maternal bacterial microbiome, thus influencing the microbiome of the infant^34, 35^. However, the actual influence of caesarean sections on infant intestinal microbiome differs among studies and is, therefore, uncertain. Moreover, the prophylactic administration of antibiotics immediately before surgery in caesarean sections, while the foetus and umbilical cord are connected, has not been considered in most studies^36, 37^. A recent study^38^ investigated the effect of exposure to cefloxime prior to caesarean section on the intestinal microbiome of infants at 10 days and 9 months of age. No distinct difference in gut microbiota composition was observed at 10 days postnatal. The nutrition method^35, 39^, presence or absence of siblings^19, 40, 41^, and gestational age^33, 42, 43^ may also influence the intestinal microbiome.

We previously examined the influence of antibiotics administered immediately before delivery on intestinal colonisation of *Bifidobacteria* in a pilot study of 1-month-old healthy Japanese infants^44^. This study showed that antibiotic administration to the mother at the time of delivery has a strong influence on the *Bifidobacteria* population and that this influence may even be more substantial than that of caesarean section.

Unlike previous studies of IAP, this pilot study was unique in a sense that the antibiotics were used for GBS and IAP in cases of premature rupture of the membranes (PROM) and routinely before caesarean section. i.e., antibiotics were used in the mother immediately before delivery, while the umbilical cord was still intact. Interestingly, the results revealed a significantly higher bifidobacterial occupancy in infants with siblings. In contrast, *Bacteroides* occupancy was substantially influenced by delivery mode compared to antibiotic exposure at delivery. As the pilot study^43^ had a cross-sectional design and was limited to 33 1-month-old infants, a continuous study comprising a larger group of infants was needed to confirm the results.

Based on the above background, the present study defined antibiotic exposure of infants through antibiotics administered to their mothers immediately before delivery as antimicrobial exposure at delivery (AED). Based on the hypothesis that AED may have a strong influence on the intestinal microbiome of healthy Japanese children in early infancy, especially on the dominant bacterial genera *Bifidobacteria* and *Bacteroides*, samples were collected from infants until 6 months after birth, and the influence of AED and other factors in the infant population were investigated.

## Results

### Subject data

A total of 142 mother and infant pairs were registered in the study, and 424 samples were obtained. Among these, 130, 127, and 126 samples collected at 1, 3, and 6 months, respectively, adhered to the inclusion criteria of the study. Subjects were excluded due to the following reasons: three premature babies dropped out by 1 month of age, one infant received antibiotic administration for fever in the neonatal period, and there were eight cases at 1 month with samples that could not be analysed. While dropouts from 1 to 3 months were due to antibiotic administration in eight cases, no samples were received in one case, and a non-analysable sample was received at 3 months in one case, dropouts from 3 to 6 months were due to antibiotic administration in one case and a non-analysable sample at 6 months in one case.

The background information of the mothers and infants at 1 month, which were influencing factors, are shown in Table 1. As the dropout cases resulted in no major change in the mean value of the background factors, the data at 3 and 6 months are shown in Supplementary Table S1 online. Antibiotics were used immediately before delivery in about 55% of cases at each age. caesarean section, GBS-positive, and PROM cases accounted for about 20%, 15%, and 15% of all cases at each age, respectively. The PROM cases included one emergency and 6 GBS-positive caesarean sections. These seven cases were included in the caesarean section group because cefazolin (CEZ) was administered as an antibiotic. In 5 PROM cases, delivery progressed rapidly, and no antibiotic was used. Of the infants born by caesarean section, the Apgar score at birth was low in two cases, oxygen was administered after birth for mild neonatal respiratory disorder in one case, phototherapy was performed for neonatal jaundice in one case, the mothers had diabetes in two cases, the mothers had hyperthyroidism in two cases, and one infant was admitted for respiratory syncytial virus (RSV) infection during the observation period and discharged after symptomatic treatment. Regarding the nutritional method, the infants enrolled in this study were those that were solely breastfed and those that were mix-fed, and none were fed milk only. All infants were confirmed to be healthy via a health examination at each age by physicians of the paediatrics and neonatology department.

**Table 1:** Background factors of 1-month-old infants and their mothers.

Background factors	Data
Number of infants	130
Number of females	70 (54.0%)
Gestational age at birth^a^	275.1 ± 9.3
Birth weight^a^	3038.9 ± 339.7
Maternal antimicrobial use at delivery	74 (56.9%)
Caesarean section	31 (23.8%)
Premature rupture of membrane	22 (16.9%)
Group B *Streptococcus*-positive status	33 (25.4%)
Infants with older siblings	67 (51.5%)
Exclusive Breast feeding	80 (61.5%)
Age of Mothers^a^	31.6 ± 5.1
Maternal history of allergy	55 (42.3%)
Neonatal respiratory disorder	2 (1.5%)
Neonatal jaundice	4 (3.1%)
RSV infection	3 (2.3%)
Maternal history of smoking	9 (6.9%)
Maternal history of Hyperthyroidism	2 (1.5%)
Maternal history of Diabetes Mellitus	2 (1.5%)

*RSV* respiratory syncytial virus.

^a^Gestational age at birth, birth weight, and maternal age are shown as the mean ± standard deviation. Other factors are given as the number of subjects and a percentage.

As for the sequencing data, input numeric totaled 15,957,768 reads, an average of 41,665 reads, a maximum of 261,246 reads, and a minimum of 17,884 reads. In addition, non-chimeric numeric reads totaled 11,055,110, an average of 28,864, a maximum of 180,762, and a minimum of 12,828.

**Figure 1:**
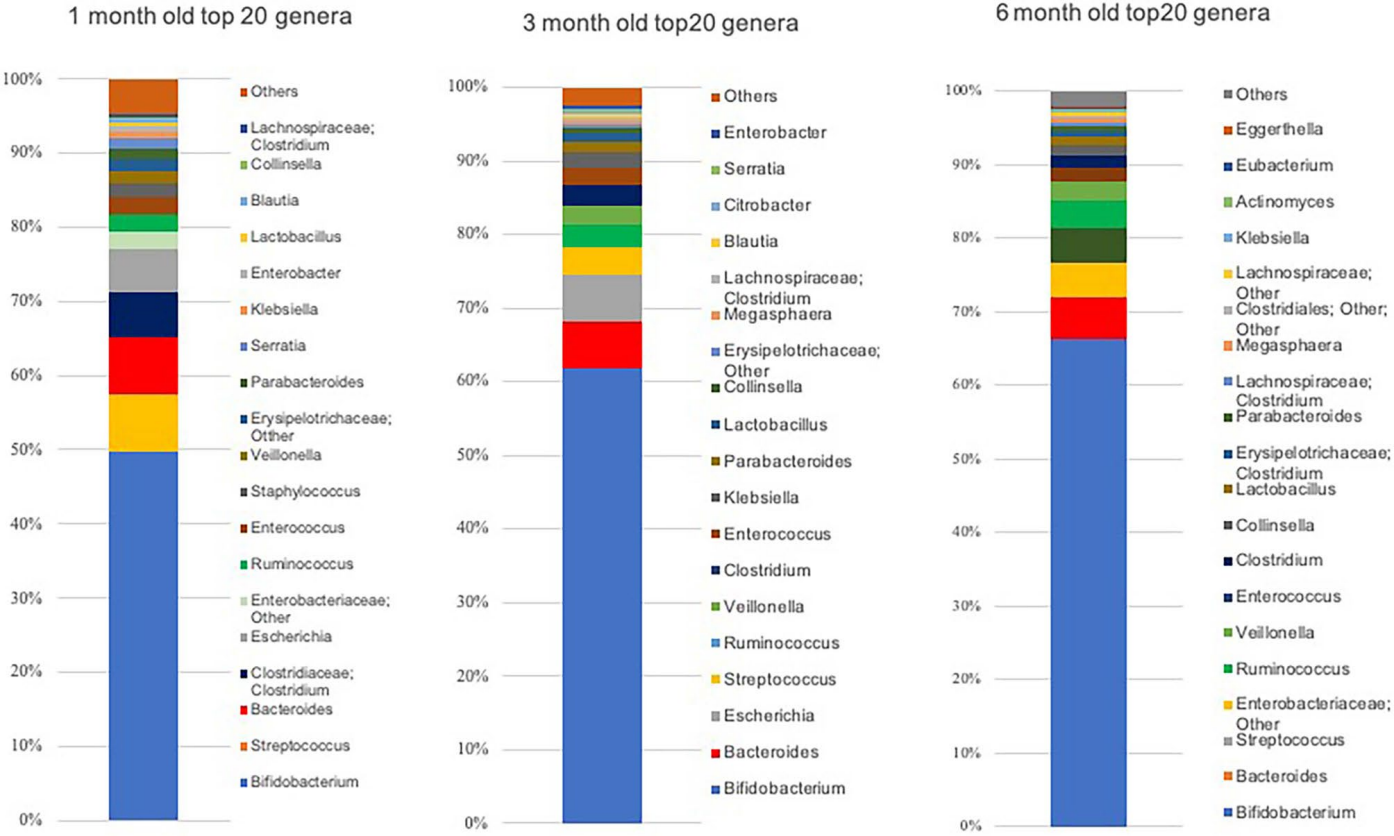
Mean occupancies of the top 20 bacterial genera constituting the intestinal microbiome at 1, 3, and 6 months after birth. At each age, the occupancies of the top 20 genera (others are indicated as ‘Others’) are shown as 100% stacked columns. The genera are shown in the order of higher occupancy from the bottom to the top. This figure was generated using Microsoft Excel for Mac 2016 (https://www.microsoft.com).

The top 20 bacterial genera constituting the intestinal microbiome at each age are shown in Fig. 1. At 1 month, based on the occupancy, the *Bifidobacteria* population was found to be overwhelmingly dominant (49.7% ± 34.1%), whereas the *Bacteroides* population was found to be the third most dominant (7.7% ± 12.6%), but its occupancy was almost equivalent to the second most dominant bacteria, *Streptococcus* (7.8% ± 12.6%). The *Bifidobacteria* population was also the most dominant at 3 months (61.7% ± 28.0%), whereas the *Bacteroides* population was the second most dominant (6.5% ± 10.9%). The *Bifidobacteria* population continued to be the most dominant at 6 months (66.2% ± 21.6%), followed by the *Bacteroides* population (5.7% ± 9.2%).

### Analysis at 1 month

The effects of background factors on the five most dominant bacterial genera of the intestinal microbiome of 1-month-old infants are shown in Table 2. *Bifidobacteria* occupancy was significantly dependent on the exposure to AED (non-AED: Odds Ratio (OR), 0.11; 95% Confidence Interval (CI), 0.03–0.39) and the existence of siblings (no sibling: OR 3.03; 95% CI 1.09–8.4), whereas that of *Bacteroides* significantly depended on the delivery method (vaginal delivery: OR 0.03; 95% CI 0.003–0.23), but not on the exposure to AED or the existence of siblings. The fourth dominant genera, *Clostridium* (6.2% ± 15.5%), showed significant effects of the exposure to AED (non-AED: OR 4.98; 95% CI 1.69–14.7), delivery method (vaginal delivery: OR 4.94; 95% CI 1.1–22.2), the existence of siblings (no sibling: OR 0.22; 95% CI 0.07–0.66), and feeding methods (exclusive breastfeeding: OR 5.88; 95% CI 2.24–15.4).

**Table 2:** Influence of background factors of the mother and child at 1 month on occupancies of the five most dominant bacterial genera in the intestinal microbiome of the infant.

Variances	*Bifidobacterium*				*Streptococcus*			*Bacteroides*				*Clostridium*				*Escherichia*			
	Odds Ratio	95% CI			Odds Ratio	95% CI		Odds Ratio	95% CI			Odds Ratio	95% CI			Odds Ratio	95% CI		
Gestational days^a^																			
1	0.96	0.91	1.02		1.05	0.99	1.11	0.93	0.87	0.98	*	1.09	1.03	1.14	**	0.92	0.87	0.98	**
2	1.03	0.97	1.09		1.03	0.98	1.09	0.89	0.83	0.94	**	1.06	1.002	1.13	*	0.93	0.88	0.99	*
3	0.95	0.90	1.00		1.01	0.96	1.07	1.00	0.95	1.07		1.03	0.97	1.10		0.96	0.91	1.02	
Birth weight^a^																			
1	0.999	0.998	1.00		1.001	0.99	1.002	0.999	0.998	1.00		1.002	1.00	1.003		0.998	0.996	0.999	**
2	0.999	0.998	1.00		1.001	0.99	1.002	0.998	0.99	1.00		1.001	1.00	1.003		0.998	0.99	1.00	
3	0.999	0.998	1.00		0.999	0.99	1.001	0.999	0.99	1.00		1.001	1.00	1.003		0.999	0.998	1.001	
Age of mothers^a^																			
1	1.00	0.91	1.11		1.02	0.93	1.13	1.09	0.94	1.14		0.91	0.83	0.99	*	1.07	0.97	1.18	
2	0.96	0.87	1.06		1.00	0.91	1.11	1.03	0.94	1.14		0.94	0.85	1.04		1.13	1.02	1.25	
3	0.98	0.89	1.08		1.03	0.94	1.14	1.10	1.00	1.21		0.96	0.86	1.07		1.05	0.96	1.16	
Male	1.47	0.57	3.75		1.20	0.57	2.53	1.35	0.56	3.23		0.85	0.33	2.18		1.45	0.67	3.14	
Vaginal delivery	1.84	0.49	6.92		1.06	0.32	3.54	0.026	0.003	0.23	**	4.94	1.10	22.23	*	0.48	0.14	1.68	
Infants without siblings	3.03	1.09	8.43	*	1.19	0.56	2.57	0.96	0.40	2.33		0.22	0.072	0.66	**	0.84	0.38	1.87	
Non-AED	0.11	0.03	0.39	***	1.22	0.53	2.82	0.65	0.27	1.56		4.98	1.69	14.69	**	0.75	0.32	1.77	
Mothers without allergy	1.33	0.51	3.50		1.27	0.60	2.72	1.14	0.46	2.78		0.85	0.33	2.23		1.62	0.73	3.58	
Exclusively breast-fed	1.06	0.41	2.73		2.06	0.96	4.41	1.61	0.66	3.94		5.88	2.24	15.43	***	0.97	0.44	2.13	

The bacterial genera are shown from left to right in the order of higher occupancy (mean). For the continuous variables (^a^ gestational age at birth, birth weight, and maternal age), occupancy was classified from 1 to 4 in ascending order from low occupancy with the occupancy of each bacterial species rounded to four decimal places. For the high occupancy group (group 4), a multinomial logistic regression analysis was performed as the category standard and the occupancy was classified into two groups based on the median for each genus for the logistic regression analysis for categorical variables. The odds ratio and 95% confidence interval were calculated using the logistic regression analysis and the multinomial regression analysis. The significance level was set at 5%. *p < 0.05, **p < 0.01, ***p < 0.001.

### Analysis at 3 months

The effects of background factors on the five most dominant bacterial genera and other high-ranking genera of the intestinal microbiome of 3-month-old infants are shown in Supplementary Table S2 online. *Bifidobacteria* occupancy was significantly dependent on the exposure to AED (non-AED: OR 0.3; 95% CI 0.09–0.9) and the existence of siblings (no sibling: OR 3.73; 95% CI 1.1–12.6), similar to that seen at 1 month (*p* < 0.05), whereas that of *Bacteroides* was significantly dependent on the delivery method (vaginal delivery: OR 0.14; 95% CI 0.03–0.63), also similar to that at 1 month (*p* < 0.05). The fifth dominant genus, *Ruminococcus* (2.9% ± 8.5%), showed a significant dependence on feeding methods (exclusive breastfeeding: OR 2.5; 95% CI 1.13–5.62).

### Analysis at 6 months

The effects of background factors on the five most dominant bacterial genera and on other high-ranking genera of the intestinal microbiome in 6-month-old infants are shown in Supplementary Table S2 online. *Bifidobacteria* occupancy showed no significant dependence on any factor, and *Bacteroides* only showed a significant dependence on exclusive breastfeeding (*p* < 0.05). There were significant effects of the delivery method (vaginal delivery: OR 0.26; 95% CI 0.07–0.91) and the existence of siblings (no sibling: OR 0.26; 95% CI 0.07–0.91) on the third most dominant genus, *Streptococcus* (4.8% ± 6.9%). The mothers’ allergy history (with history of allergy: OR 2.67; 95% CI 1.20–5.94) significantly affected the fourth most dominant genus, *Enterobacteriaceae*; and feeding methods (exclusive breastfeeding: OR 0.29; 95% CI 0.12–0.66) significantly affected the fifth most dominant genus, *Ruminococcus*.

### Effects of AED

Infants at each age were divided based on the AED status and delivery method, and background factors of the mother and child were compared. The results are shown in Supplementary Table S3 online. In infants born via caesarean section, the existence of siblings and age of the mother were significantly higher due to the influence of the previous caesarean section, and the gestational age and birth weight were significantly lower because the date of caesarean delivery was decided beforehand unless performed as an emergency. This tendency also existed between the two types of delivery methods in the AED group, but a sub-group analysis of the vaginal delivery group, excluding the influence of caesarean section, showed no difference in background factors between the AED and non-AED groups. The effect of background factors that influenced the dominant bacterial genera at each age (AED, delivery method, siblings) and the occupancies by *Bifidobacteria* and *Bacteroides* were then analysed.

**Figure 2:**
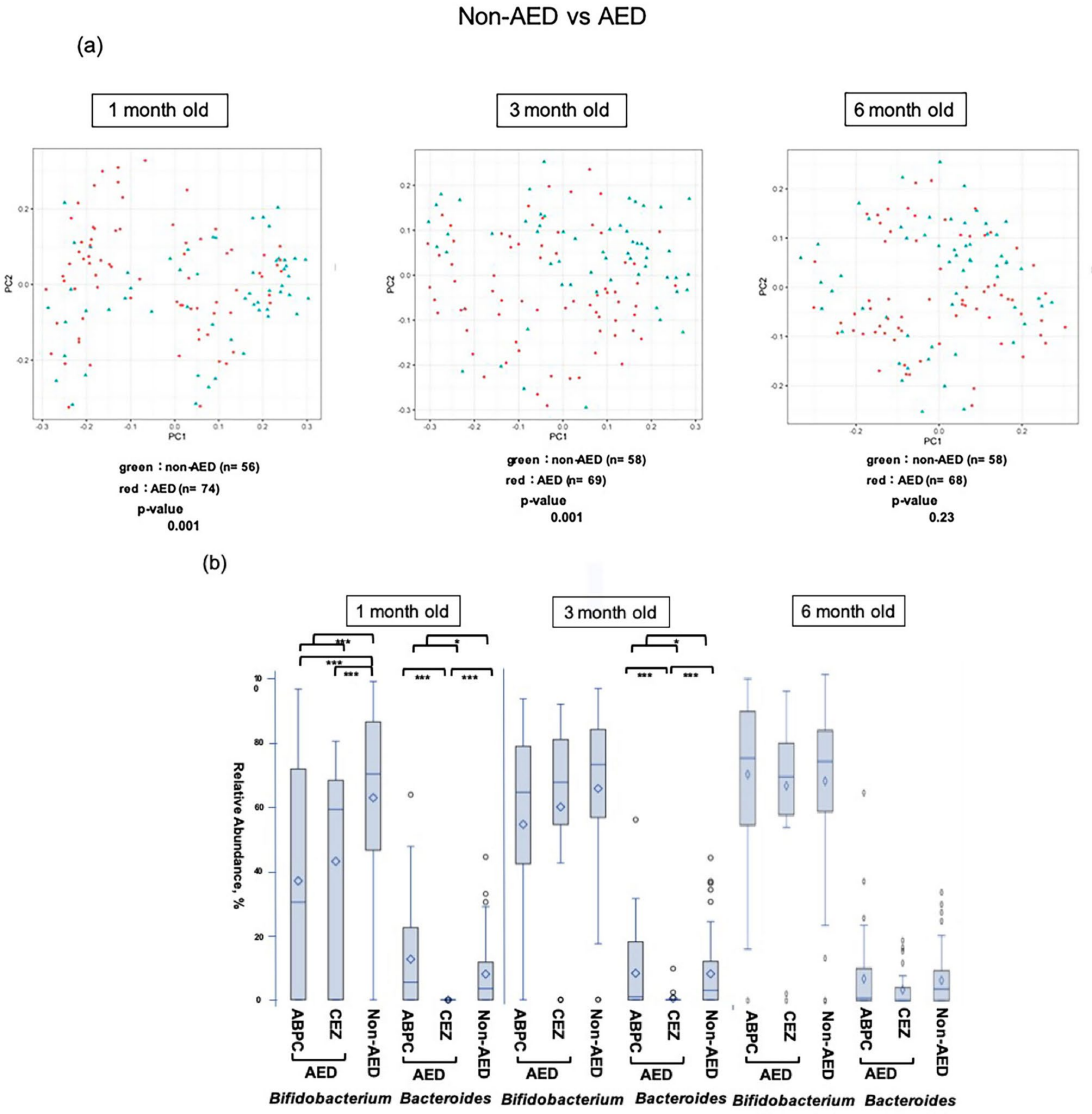
Comparison of the β diversity and occupancy of the top two bacteria species in the AED and non-AED groups. (**a**) Comparison of β diversity of the intestinal microbiome between infants at each age with (AED) and without (non-AED) antibiotic exposure at delivery. (**b**) *Bifidobacteria* and *Bacteroides* occupancies in infants in the AED and non-AED groups, shown as box-whisker plots at each age. The AED group was also divided into ampicillin-treated (ABPC) and cefazolin-treated (CEZ) groups. Comparison of occupancy among these groups and the non-AED group was performed using Bonferroni multiple comparison test. Comparison between the AED and non-AED groups was performed using Mann–Whitney U-test. The ABPC, CEZ, and non-AED groups included 43, 31, and 56 infants at 1 month (130 in total); 42, 27, and 58 infants at 3 months (127 in total); and 40, 28, and 58 infants at 6 months (126 in total). The significance level was set at 5%. *p < 0.05, ***p < 0.001 This figure was generated using SAS version 9.4 (https://www.sas.com).

AED had a significant effect on the overall diversity of the intestinal microbiome at 1 and 3 months (Fig. 2a). *Bifidobacteria* occupancy was significantly lower in AED cases than in non-AED cases in 1-month-old infants, regardless of the use of ampicillin (ABPC) or CEZ (*p* < 0.001). In contrast, in 3-month-old infants, occupancy was not affected by AED. The *Bacteroides* population was markedly lower in the CEZ group at both 1 and 3 months than in the non-AED group (*p* < 0.001) and this tendency was also noted in the ABPC group. A significant difference was also found between AED and the non-AED groups (*p* < 0.05) (Fig. 2b).

**Figure 3:**
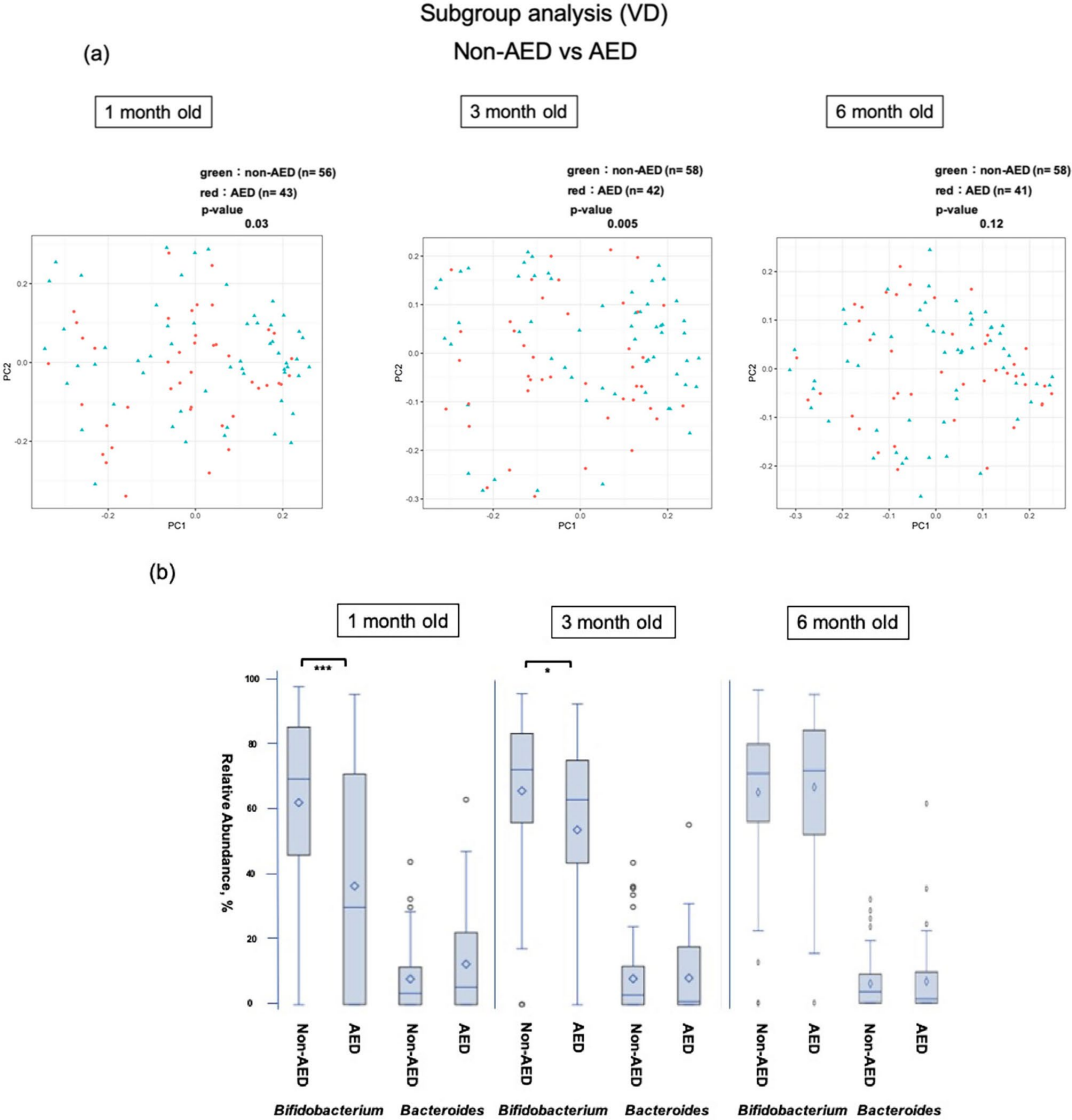
Comparison of the β diversity and occupancy of the top two bacteria species in the AED and non-AED groups of the vaginal delivery group. (**a**) Comparison of β diversity of the intestinal microbiome between infants at each age with (AED) and without (non-AED) antibiotic exposure at vaginal delivery. (**b**) Comparison of *Bifidobacteria* and *Bacteroides* occupancies in infants in the AED and non-AED groups with vaginal delivery, shown as box-whisker plots at each age. The AED and non-AED groups included 43 and 56 infants at 1 month (99 in total), 42 and 58 infants at 3 months (100 in total), and 41 and 58 infants at 6 months (99 in total). The significance level was set at 5%. *p < 0.05, ***p < 0.001 using Mann–Whitney U-test. This figure was generated using SAS version 9.4 (https://www.sas.com).

In a sub-group analysis of AED in vaginal delivery cases without CEZ administration (i.e., excluding infants born by caesarean section), there was a significant difference in diversity (*p* = 0.03) (Fig. 3a). *Bifidobacteria* occupancy in 1-month-old infants was significantly lower in the AED group (all were included in the ABPC group) (*p* < 0.001), and a significant difference was also noted at 3 months (*p* < 0.05). In contrast, occupancy of *Bacteroides* did not differ between these two groups (Fig. 3b). In the AED group, the exposure to antibiotics did not affect the *Bifidobacteria* and *Bacteroides* populations in the PROM and GBS-positive groups (Supplementary Fig. S1 online).

### Effects of the delivery method

**Figure 4:**
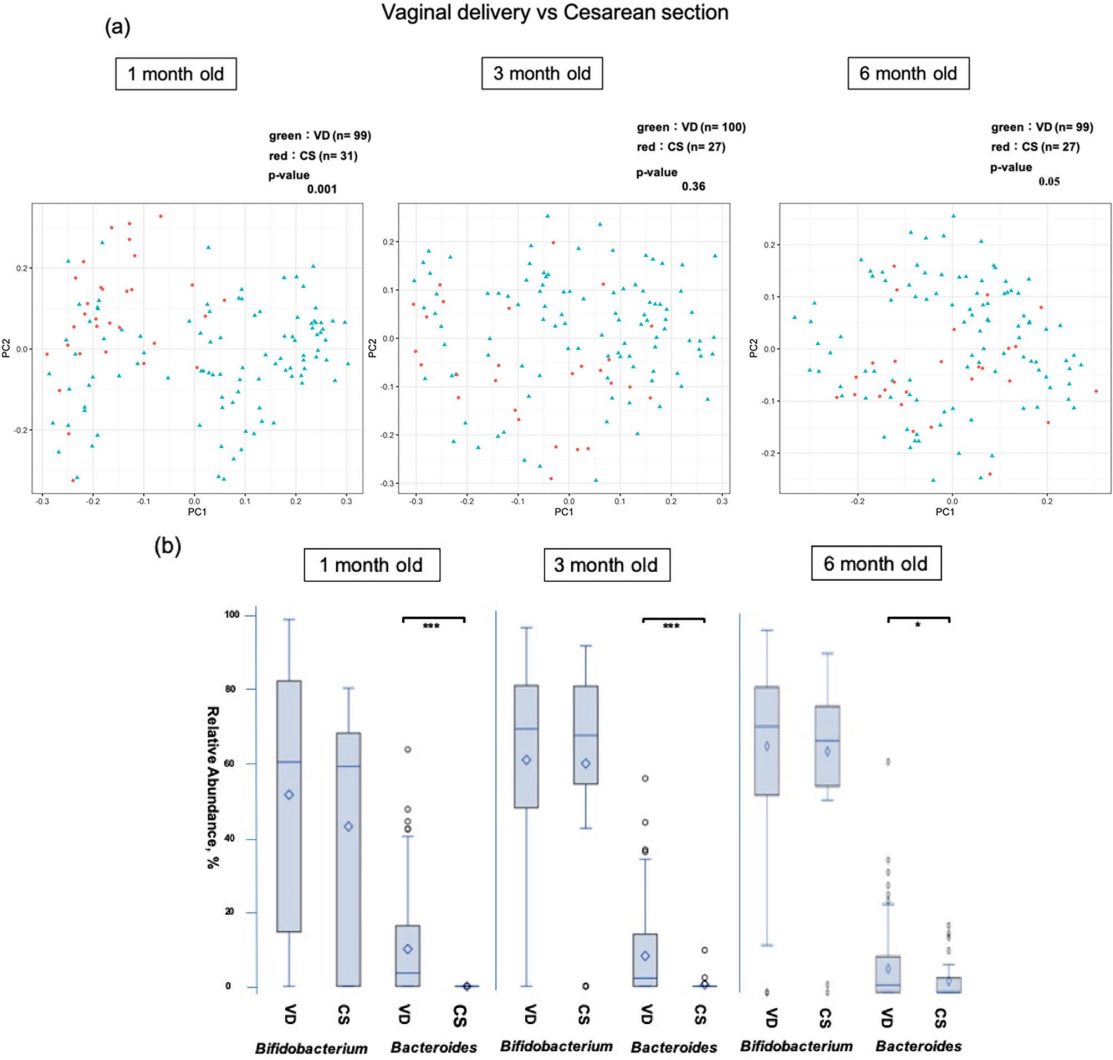
Comparison of the β diversity and occupancy of the top two bacteria species in the vaginal delivery (VD) and caesarean section (CS) groups. (**a**) Comparison of β diversity of the intestinal microbiome at each age between vaginal delivery (VD) and caesarean section (CS) (**b**) *Bifidobacteria* and *Bacteroides* occupancies between delivery methods, shown as box-whisker plots at each age. The VD and CS groups included 99 and 31 infants at 1 month (130 in total), 100 and 27 infants at 3 months (127 in total), and 99 and 27 infants at 6 months (126 in total). The significance level was set at 5%. *p < 0.05, ***p < 0.001 using Mann–Whitney U-test. This figure was generated using SAS version 9.4 (https://www.sas.com).

**Figure 5:**
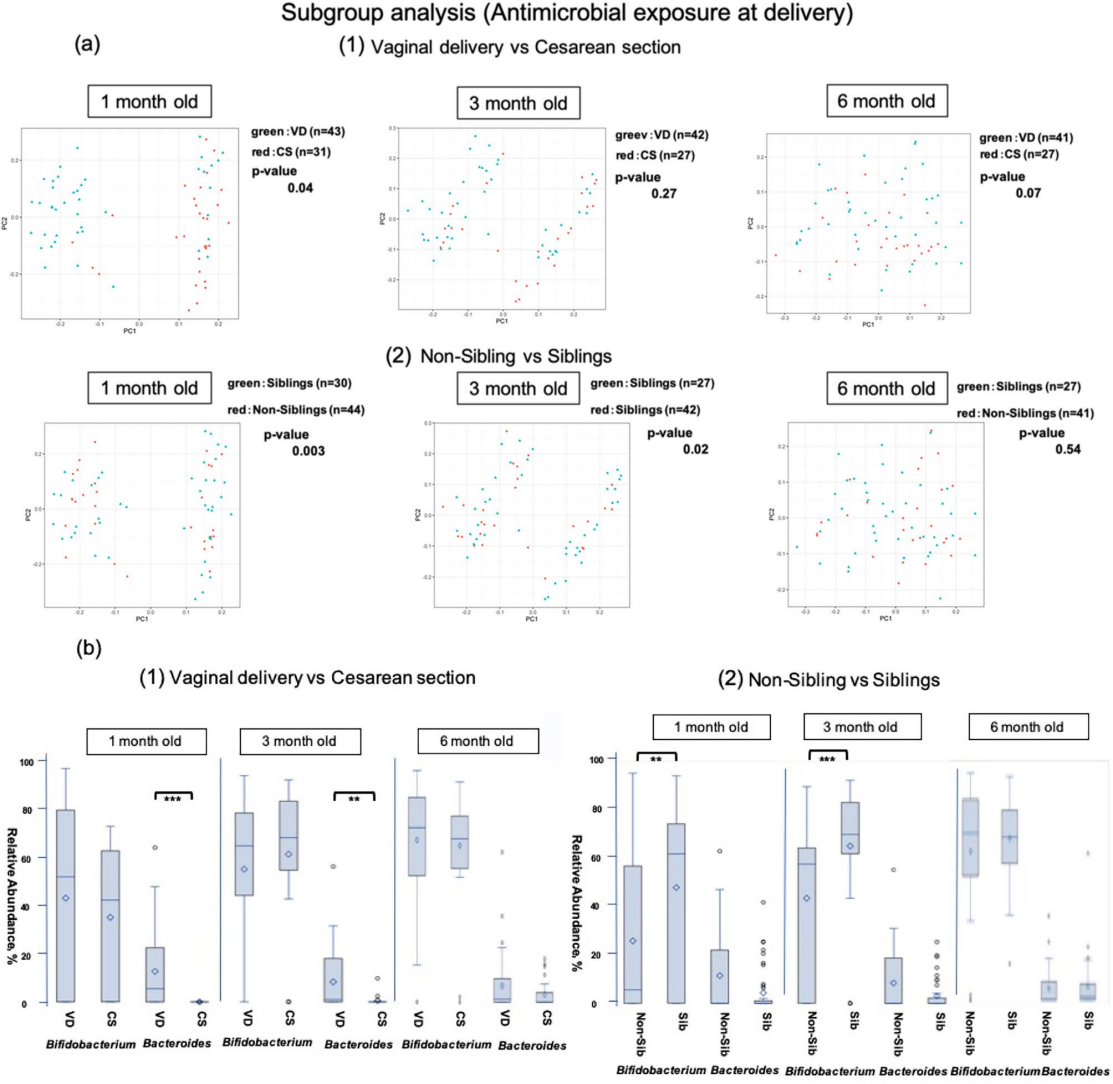
Comparison of the β diversity and occupancy of the top two bacteria species in the vaginal delivery (VD) and caesarean section (CS) groups of the AED group. (**a**) Comparison of β diversity of the intestinal microbiome at each age between delivery methods (1) and with and without siblings (2) in the AED group. VD: vaginal delivery, CS: caesarean section, Non-Siblings: infants without a sibling, Siblings: infants with older siblings. (**b**) *Bifidobacteria* and *Bacteroides* occupancies in the AED group compared between delivery methods (1) and presence of absence of siblings (2), shown as box-whisker plots at each age. (1) The vaginal delivery (VD) and caesarean section (CS) groups included 43 and 31 infants at 1 month (74 in total); 42 and 27 infants at 3 months (69 in total), and 42 and 27 infants at 6 months (68 in total). (2) The Sib and non-Sib groups included 44 and 30 infants at 1 month (74 in total), 42 and 27 infants at 3 months (69 in total), and 41 and 27 infants at 6 months (68 in total). The significance level was set at 5%. **p < 0.01, ***p < 0.001 using Mann–Whitney U-test. This figure was generated using SAS version 9.4 (https://www.sas.com).

The delivery method significantly influenced the diversity of the intestinal microbiome at 1 month (Fig. 4a). The occupancy of *Bifidobacteria* did not differ with age, whereas that of *Bacteroides* was significantly lower in 1- and 3-month-old infants born via caesarean section (*p* < 0.001) (Fig. 4b). A comparison of delivery methods within the AED group gave similar findings (Fig. 5a,b).

### Effects of siblings

**Figure 6:**
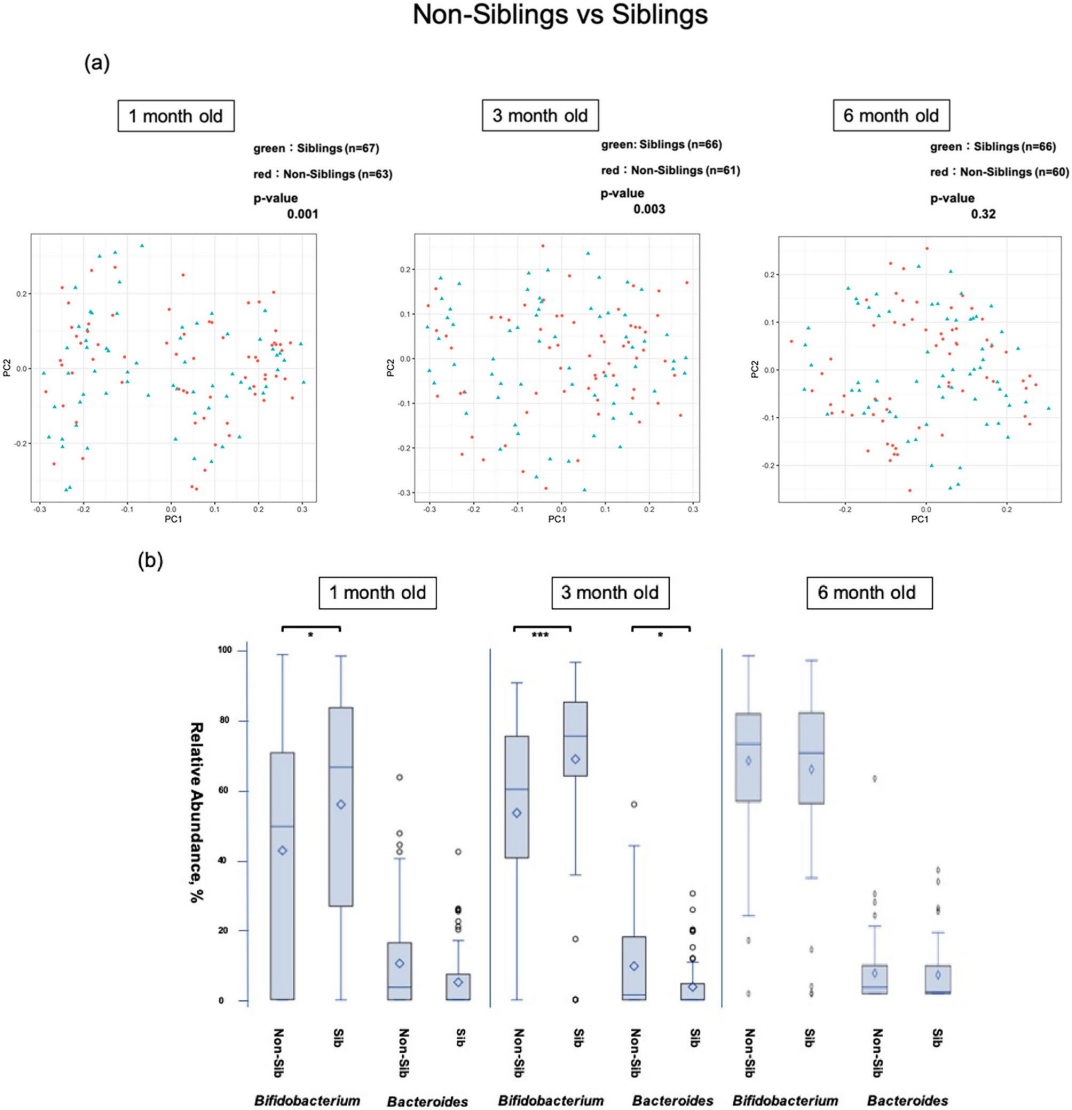
Comparison of the β diversity and occupancy of the top two bacteria species between the infants with siblings and those without siblings (**a**) Comparison of β diversity of the intestinal microbiome between infants with and without siblings. (**b**) *Bifidobacteria* and *Bacteroides* occupancies between infants with siblings (Sib) and without siblings (Non-Sib), shown as box-whisker plots at each age. The Sib and non-Sib groups included 67 and 63 infants at 1 month (130 in total), 61 and 66 infants at 3 months (127 in total), and 66 and 60 infants at 6 months (126 in total). The significance level was set at 5%. *p < 0.05, ***p < 0.001 using Mann–Whitney U-test. This figure was generated using SAS version 9.4 (https://www.sas.com).

The presence of siblings significantly changed the diversity of the intestinal microbiome at 1 and 3 months (Fig. 6a). *Bifidobacteria* occupancy was significantly higher in 1-month-old (*p* = 0.001) and 3-month-old (*p* < 0.001) infants with siblings. Occupancy of *Bacteroides* did not differ at 1 month but was significantly lower in infants with siblings at 3 months (*p* < 0.05). At 6 months, there was no significant difference in occupancy for either genus (Fig. 6b). Sub-group analysis within the AED group also showed a significant change in the diversity of the intestinal microbiome at 1 and 3 months (Fig. 5a), and bifidobacterial occupancy was significantly higher in 1- and 3-month-old infants with siblings (*p* < 0.01 and *p* < 0.001, respectively) (Fig. 5b).

### Time-course changes in Bifidobacteria and Bacteroides (linear mixed-effect model)

**Figure 7:**
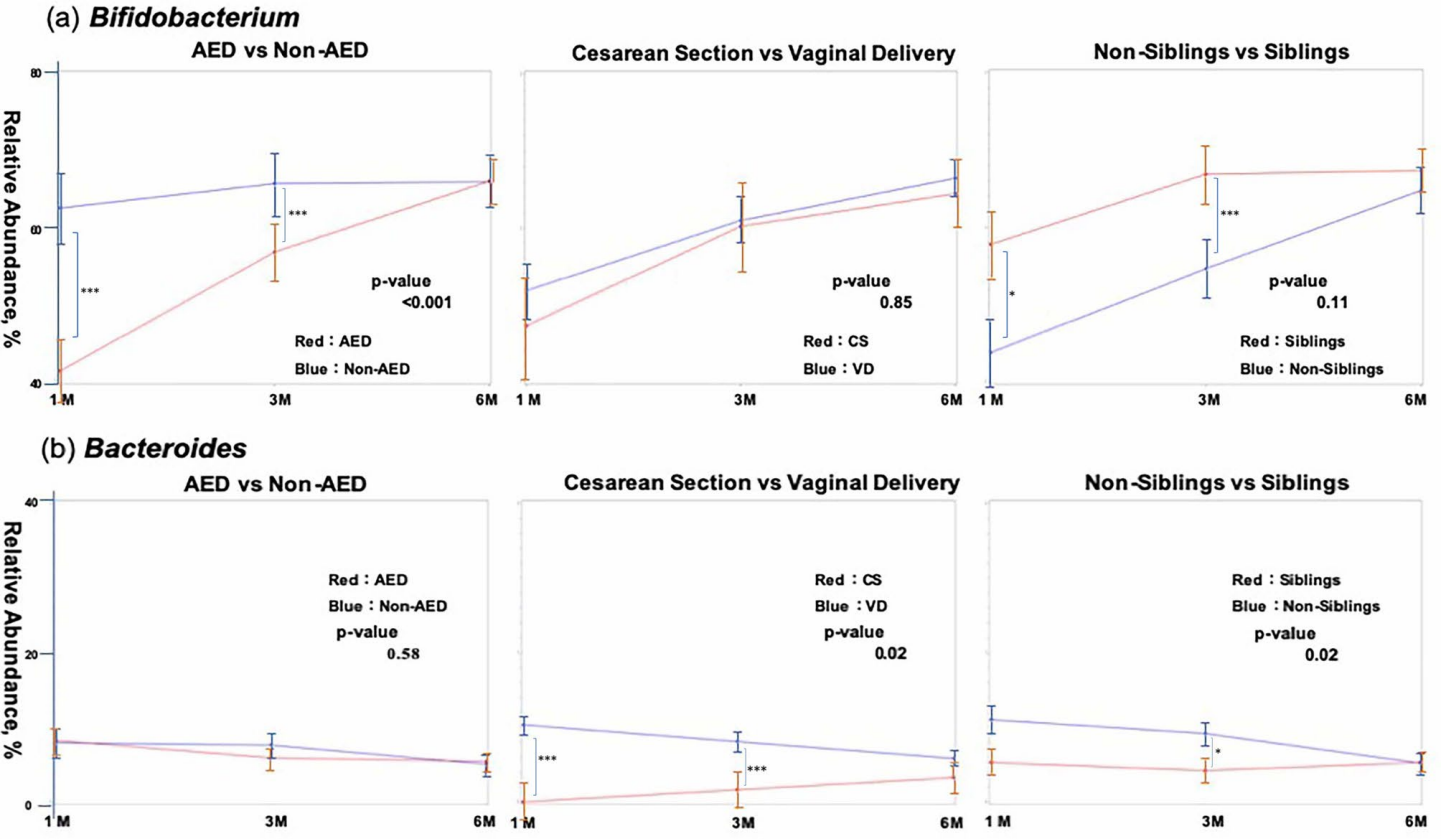
Time-course changes in bifidobacterial (**a**) and *Bacteroides* (**b**) occupancies (from 1 to 6 months after birth) based on AED or non-AED, delivery method, and the presence or absence of siblings, using a linear mixed-effect model. The analysis set at 1 month included 130 infants. Dropouts at 3 and 6 months were handled as missing values. The standard deviation by age in months is shown in bar form in each figure. Significant differences in occupancies between the two groups at each time point are also indicated in each comparison. The significance level was set at 5%. *p < 0.05, ***p < 0.001 using the Mann–Whitney U-test. This figure was generated using SAS version 9.4 (https://www.sas.com).

Comparison of bifidobacterial occupancy in AED and non-AED infants using a linear mixed-effects model (Fig. 7a) showed a significant difference over 6 months after birth, with lower occupancy in the AED group, but the difference in occupancy decreased with time. There was, however, no difference seen due to the delivery method. Occupancy appeared to be higher in infants with siblings, but there was no significant difference by the sixth month. Occupancy of *Bacteroides* did not differ significantly between the AED and non-AED groups (Fig. 7b), but the occupancy was lower in the caesarean section group and in infants with siblings.

## Discussion

The results of this study indicated that the diversity of the intestinal microbiome was influenced by AED, delivery method, and siblings, with significant effects on *Bifidobacteria* and *Bacteroides* populations, which remained dominant at a combined occupancy of 60–70% in infants aged up to 6 months. These findings confirm the results of an earlier pilot study^44^ in 1-month-old infants.

AED to β-lactamase antibiotics has a major influence on *Bifidobacteria* population in early infants regardless of the use of ABPC or CEZ, with the influence being especially marked in 1-month-old infants. The influence of ABPC persisted until 3 months, but then gradually weakened and mostly disappeared by the sixth month. These results also conform with those of the pilot study^44^. Several previous studies have suggested a minor effect of IAP on the *Bifidobacteria* occupancy. However, these studies were limited to use of antibiotics for specific reasons, such as for GBS-positive mothers^45, 46^ or in the late stage of delivery^30^, and the study design and screening timeline of the intestinal microbiome were inconsistent. Although there has been a previous study^38^ on the effect of cefuroxime administration to mothers on the gut composition of infants immediately after delivery (10 days) and after weaning (9 months), the present study is the first, to the best of our knowledge, to evaluate the effects of antibiotic administration to mothers immediately before delivery on 1-, 3-, and 6-month-old infants, including the cases of caesarean section. The study comprised a statistically appropriate number of samples, thereby allowing sub-group analyses. The effects were evaluated until 6 months after birth using a 16S rRNA-targeting next-generation sequencer.

The delivery method (vaginal vs caesarean section) did not affect the *Bifidobacteria* occupancy in 1-month old infants, but there was a significant difference in the CEZ and non-AED groups. The delivery method did affect the *Bifidobacteria* population between the ABPC and CEZ groups. Thus, *Bifidobacteria* occupancy was significantly influenced in the caesarean section and CEZ groups compared to non-AED infants, but this effect did not change due to AED in the vaginal delivery group (i.e., the ABPC group). These results suggest that β-lactam antibiotics may directly influence bifidobacterial occupancy. Influence of the delivery method on *Bifidobacteria* population from immediately after birth to early infancy has been shown previously^40, 47–50^, but the influence of antibiotics administered before caesarean section^36, 37, 51^ was not considered in these studies, and this effect may have been simultaneously observed. The present study is not capable of judging whether the observed effect is due to the infant not passing through the birth canal in caesarean section or CEZ. Further studies with the use of the same antibiotics for vaginal delivery and caesarean section are therefore warranted.

*Bifidobacteria* colonisation was also influenced by the presence or absence of siblings. *Bifidobacteria* occupancy in AED infants was significantly higher in those with siblings. The results suggested that at least until 3 months after birth, the presence of an elder sibling promoted the colonisation of *Bifidobacteria*, even in infants exposed to antibiotics at delivery, i.e., there may be mutual interference of the microbiome between siblings. This may explain the maintenance of high *Bifidobacteria* occupancy in infants, even in the AED group. In a previous pilot study^44^, the presence of siblings was suggested to influence IAP, and the present study confirmed this effect. Previous studies on the effects of siblings have reported that *Bifidobacteria* colonisation occurs more easily in infants with siblings^19, 40, 41^. However, this effect has been scarcely studied as compared to studies on factors such as AED and the delivery method, particularly in Japanese infants. Thus, the present study is significant in showing the effect of siblings in a large cohort study. This effect was confirmed using a sub-group analysis within the AED group to allow interpretation in the context of AED. The association of this effect with the intestinal microbiome of siblings requires a continuous study, including siblings living together.

The influence of the caesarean section was more substantial than that of AED in *Bacteroides*. This effect was firmly maintained at 3 months and persisted at least until 6 months after birth. The same tendency was observed in the sub-analysis of the AED group. This confirmed that birth via caesarean section is an important factor in the occupancy of *Bacteroides*, compared to AED. A similar persistence of the influence of the caesarean section on *Bacteroides* until the weaning period and thereafter has been previously reported^34, 40, 50, 52, 53^. However, as described above, all caesarean section cases received preoperative CEZ. Thus, although the *Bacteroides* population was not influenced by ABPC, it may have been markedly influenced by CEZ. This possibility should be examined in future studies.

The *Bacteroides* population was not affected by siblings at 1 month, but a significant effect was seen at 3 months, with no influence at 6 months. In contrast to *Bifidobacteria*, siblings negatively influenced the occupancy of *Bacteroides*. The influence of siblings on the *Bacteroides* population has been shown before^40^, with the occupancy by *Bacteroides* at 18 months after birth being higher in infants with siblings, in contrast to the findings of this study until 6 months of age^54^. Similar to the effect seen on *Bifidobacteria*, the effect on *Bacteroides* until 3 months may reflect mutual interference among siblings.

Many studies have examined the clinical significance of colonisation of the intestinal microbiome with *Bifidobacteria* and *Bacteroides* in early infancy^17–22^. The present study evaluated the influence of AED on early infants in terms of the effects on their subsequent health. The samples from this study were also used to assess the relation of allergies with changes in the intestinal microbiome. This study was performed as a part of a cohort study evaluating the clinical significance of changes in the intestinal microbiome during early infancy in healthy Japanese infants and their effect on the intestinal microbiome later in life. Data at 1, 3, and 6 months after birth were used in this study, but data related to longer-term time-course changes in the same individuals are needed for a complete and comprehensive investigation. The composition of the intestinal microbiome shows substantial similarities at different time-points in the same individual^55^, but external factors influencing the intestinal microbiome increase with growth, such as the increase in baby food intake, interaction with siblings and other infants in group nursing, and further use of antibiotics for various diseases. However, this study is significant as a cohort study with a statistically significant large number of samples of the intestinal microbiome of infants in the first 6 months of life, which is a crucial period for the development of the immune system as this is a time at which external factors have the least influence in the entire lifetime^24–26^.

Despite its importance, the present study is not without limitations. With regard to the possibility of the AED and differences in delivery modes in this study affecting *Bifidobacterium* and *Bacteroides*, a strong “relationship” was observed. However, this study is a prospective cohort follow-up observation study, and comparative experimental studies are needed to confirm more robust causal relationship, together with follow-up observations. This study was conducted in a single hospital, which does not allow for extrapolation of the obtained results to the population from other regions within Japan and beyond, with differences in ethnicity or geography. Although the protocol for the use of antimicrobial agents before the delivery was similar to that in previous studies on IAP^27, 29^, multi-centre studies are needed for validation of such extrapolations. Additionally, while this study focused on several factors that could have a significant impact on the gut microbiota of infants, it did not account for some factors such as feeding methods as infants fed solely on milk were not included; thus, comparison of the feeding methods is not adequate. These points will likely cause a selection bias, which could affect colonisation characteristics of certain bacteria in the intestinal microbiota of infants. Inclusion of another group of infants that were milk-fed for the most part along with top feed is imperative to understand the factors influencing gut microbiota in infants. Moreover, occupancy was used in the comparison of bacteria in this study. A qualitative assessment based on qPCR is required to accurately assess the effect of factors such as AED and delivery method, rather than a relative assessment based on occupancy as was done in this study^56, 57^.

## Conclusions

This prospective cohort study confirmed the findings from a previous pilot study indicating that β-lactam antibiotics administered to mothers immediately before delivery significantly influence the intestinal microbiome of healthy Japanese infants at 1 and 3 months after birth. This effect is more substantial than the effect of the delivery method on the dominant bacterial genus *Bifidobacterium*. The evaluation of the influence of AED requires the inclusion of all antibiotics used immediately before delivery, including before the caesarean section. The presence of siblings also affects *Bifidobacteria* colonisation, and this effect persists until 3 months and increases with time. In contrast, for *Bacteroides*, the influence of the delivery method is greater than that of AED. However, it is unclear whether this is an influence of delivery via caesarean section or CEZ administration. These results provide a new perspective on essential factors influencing the intestinal microbiome in early infancy, which is vital for the development of intestinal immunity. The clinical significance of the results in the later life of the infants requires further long-term studies.

## Methods

### Ethics approval and consent to participate

All experimental procedures used in the study complied with the ethical standards of national guidelines of the Japanese government on human experimentation and the Declaration of Helsinki 1975, as revised in 2008. The study was approved by the institutional review boards of Iwate Prefectural Iwai Hospital (No. 1234) and Juntendo University (No. 2017127). Written informed consent for participation and publication was obtained from all mothers.

### Subjects

This prospective cohort study was performed as a part of a study investigating the association of allergic diseases with time-course changes observed in the microbiome in infants. The subjects included 142 infants and their mothers, who gave consent to registration before delivery or 2 weeks after delivery at Iwate Prefectural Iwai Hospital between February 2018 and March 2019. This hospital is in the Tohoku (Northeast) region of Japan and handles about 800 deliveries each year as a core perinatal medical care centre in the southern Iwate prefecture.

Inclusion criteria were as follows: infants born at full-term with natural delivery or caesarean section, whose mothers had not been exposed to antibiotics for 1 month before delivery, except for the antimicrobial used just before the delivery. Faecal samples were collected at 1, 3, and 6 months after birth by the parents.

Exclusion criteria were as follows: premature babies born before 37 weeks of gestation; if the babies received any antibiotics during first 6 months from their birth, the samples collected were handled as dropouts. If the sample collection was missed, the infant was considered as a dropout at that age and after that. If a sample was inappropriate for MiSeq analysis and could not be analysed, data for this infant were excluded from the analysis only at this time-point, whereas the analytical results for the infant at all other ages were used.

During IAP at delivery, a single dose of CEZ (1 g) was given systemically to mothers for all caesarean sections just before the surgery. ABPC (2 g) was given at least 4 h before delivery, followed by intermittent administration every 6 h until delivery in GBS-positive and PROM cases. Antibiotics were administered to all subjects at the dose and time defined in the clinical protocol determined by the hospital board.

Containers for collection of faecal samples at 1 month after birth were handed to mothers during hospitalisation for delivery or at the infant health examination 2 weeks after delivery. The container for the collection of faecal samples at 3 months was sent by mail to the address of each registered infant from Core Technology Laboratories, Asahi Group Holdings, and the container for 6 months was sent by mail from the Department of Microbiome Research, Juntendo University. Each faecal sample collected by the infants' parents was transferred to a test tube (Techno Suruga Laboratory, Shizuoka, Japan) containing 0.001% bromothymol and 100 mM Tris–HCl (pH 9), 40 mM EDTA, 4 M guanidine thiocyanate, and was mixed well as described in a previous study^58^. Mixed faecal samples were delivered to a laboratory of Asahi Group Holdings (Sagamihara, Kanagawa, Japan) within one or two days from Ichinoseki city, Iwate prefecture, where most of the enrolled infants resided (the distance between the two locations is approximately 480 km), and were stored at − 80 °C until processing for DNA extraction. The test tubes containing the samples were dissolved in a solution (provided in the kit mentioned above), which ensured that the composition of the intestinal microbiome present within the body was stable even at room temperature^58^.

### DNA extraction

The processed samples were subjected to DNA extraction, as described previously^44^. Briefly, the samples (2 mL) were transferred to plastic tubes, centrifuged at 14,000× g for 3 min, washed in 1.0 mL of phosphate-buffered saline, and centrifuged at 14,000× g. Pellets were re-suspended in 500 μL of extraction buffer (166 mM Tris/HCl, 66 mM EDTA, 8.3% sodium dodecyl sulphate, pH 9.0) and 500 μL of TE buffer-saturated phenol. Next, 300 mg of zirconium beads (0.1 mm diameter) was added to the suspension, and the mixture was vortexed vigorously for 60 s × 3 times using a Multi-Beads Shocker (Yasui Kikai Corp., Osaka, Japan). After centrifugation at 14,000 × g for 5 min, 400 μL of the supernatant was purified using a Maxwell Instrument (Promega KK, Tokyo, Japan).

### Sequencing and data processing

16S rRNA gene sequencing was performed using a MiSeq V3 kit as per the manufacturer's protocol (Illumina, San Diego, CA, USA). Briefly, the V3-V4 region of the bacterial 16S rDNA was amplified using PCR with forward and reverse primers (5′-TCG GCA GCG TCA GAT GTG TAT AAG AGA CAG CCT ACG GGA GGC WGC AG-3′ and 5′-GTC TCG TGG GCT CGG AGA TGT GTA TAA GAG ACA GGA CTA CHV GGG TAT CTA ATC C-3′) with the TaKaRa Ex Taq HS Kit (TaKaRa Bio, Shiga, Japan) from 5 ng of DNA from faecal samples. After the PCR products were purified with Agencourt AMPure XP (Beckman Coulter, Indianapolis, IN, USA) and amplified using a Nextera XT Index Kit v2 (Illumina, San Diego, CA, USA). After the second round of PCR, the products were again purified using Agencourt AMPure XP. The library was quantified, normalised, and pooled in equimolar amounts. Sequencing was conducted using a paired-end 2 × 300-bp cycle run on an Illumina MiSeq system with a MiSeq Reagent Kit v.3 (600 cycles).

### 16S rRNA-based taxonomic and diversity analysis

QIIME2 (Quantitative Insights into Microbial Ecology, http://qiime2.org/) v.2019.4.0. was used for sequence analysis^59^. The sequence quality control, removing chimeric sequences and feature table construction were performed with the DADA2 plugin^60^. The primers sequence were trimmed and the remaining forward and reverse sequences were truncated to a final length of 280 bp. Phylogenetic diversity analyses were carried out with q2-phylogeny and q2-diversity, and beta diversity was visualised using principal coordinate analysis (PCoA). The feature classifiers were performed with q2-feature-classifier plugin using “gg-13-8-99-nb-classifier.qza.” from the greengenes database as reference sequences.

### Data collection

The following data were collected from medical records at Iwate Prefectural Iwai Hospital: delivery method, gender, body weight at birth, perinatal history, records of hospital visits, and treatments received by the infant up to 6 months after birth including the use of antibiotics after birth. Additional information related to age (days) at sample collection, feeding method (breastfeed exclusively or added top feed), and siblings were obtained from a questionnaire completed by the mothers. The following data about the mothers were also collected from medical records and a questionnaire: age, delivery method, history of allergies (food allergy, bronchial asthma, atopic dermatitis, allergic rhinitis), abnormal findings at delivery (including PROM and GBS-positive status), antimicrobial use during late pregnancy, and systemic antibiotics (including types) given at delivery.

### Statistical analysis

The significance of the difference between the two groups was analysed using a non-parametric ANOSIM (analysis of similarities) test based on unweighted UniFrac distances within QIIME2 (https://qiime2.org/). Acquired 16S rRNA gene data for bacteria were analysed using SAS 9.4 (SAS Institute Inc., Cary, NC, USA). Background factors of the mother and child were compared using the Mann–Whitney U-test for continuous variables and the Pearson’s chi-square test for categorical variables. The influence of each background factor on occupancies by higher-rank dominant bacterial genera in the intestinal microbiome at each age was examined using logistic regression analysis and multinomial logistic regression for categorical variables and continuous variables, respectively. The dependence of occupancy of each bacterial genus on factors, for which a significant association was found in diversity analysis and logistic regression analysis, was examined using a Mann–Whitney U-test for between-group comparison and using a Kruskal–Wallis test with a Bonferroni correction for multiple group comparison. Continuous comparative changes of dominant bacterial genera due to different factors were analysed using a linear mixed-effect model (random intercept and first-order autoregression model). The significance level was set at *p* < 0.05 in all analyses.

## Supplementary Information

Supplementary Information

## Data Availability

The data generated during the current study are available in the Figshare repository (https://doi.org/10.6084/m9.figshare.12000255), and the sequencing data is deposited in the DNA Data Bank of Japan (DDBJ; accession number: DRA010467).
