# Supplementary material for: Administration of β-lactam antibiotics and delivery method correlate with intestinal abundances of Bifidobacteria and Bacteroides in early infancy, in Japan
Source: Sci Rep. 2021 Mar 18;11:6231. doi: 10.1038/s41598-021-85670-z (PMC7973812; doi:10.1038/s41598-021-85670-z)
Supplement: Supplementary file 1 — Supplementary Information [file 41598_2021_85670_MOESM1_ESM.docx]

Administration of β-lactam antibiotics and delivery method correlate with the intestinal abundances of *Bifidobacteria* and *Bacteroides* in early infancy, in Japan

Naruaki Imoto^1*^, Chie Kano^2^, Yumi Aoyagi^2^, Hiroto Morita^2^, Fumitaka Amanuma^3^, Hidekazu Maruyama^3^, Shuko Nojiri^4^, Naoyuki Hashiguchi^5^, and Shin Watanabe^1^

^1^ Department of Microbiome Research, School of Medical Science, Juntendo University, Bunkyo Ward, Tokyo, Japan

^2^ Core Technology Laboratories, Asahi Group Holdings, Ltd., Sagamihara, Kanagawa, Japan

^3^ Department of Paediatrics, Department of Neonatology, Iwate Prefectural Iwai Hospital, Ichinoseki, Iwate, Japan

^4^Juntendo Clinical Research Support Centre, Juntendo University, Bunkyo Ward, Tokyo, Japan

^5^ Department of Emergency and Disaster Medicine, School of Medical Science, Juntendo University, Bunkyo Ward, Tokyo, Japan

**Supplementary information**


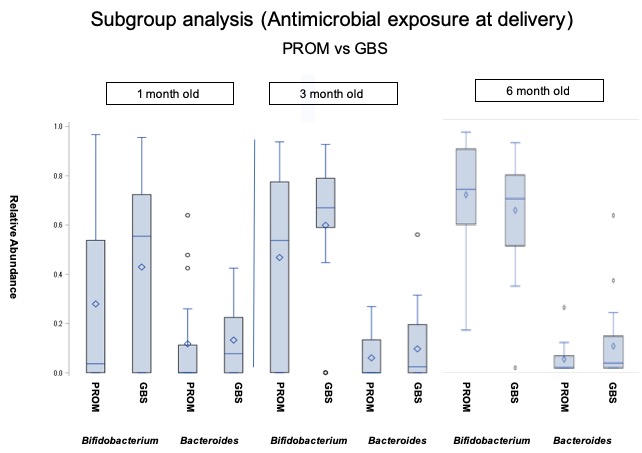


Supplementary Figure S1 online*.* *Bifidobacterium* and *Bacteroides* occupancies of premature rupture of membrane (PROM) or Group B *Streptococcus* (GBS) positive cases in the vaginal delivery group within the antimicrobial exposure at delivery (AED) group, shown as box-whisker plots at each age. For comparison of the occupancy between groups with and without siblings, a Mann–Whitney U-test was used. The PROM and GBS groups included 22 and 33 infants at 1 month (130 in total), 19 and 26 infants at 3 months (127 in total), and 21 and 30 infants at 6 months (126 in total) of age, respectively. The significance level was set at 5%. No significant difference due to AED was found for *Bifidobacterium* or *Bacteroides* occupancy at any age.

Supplementary Table S1 online. Background factors of 3- and 6-month-old infants and their mothers.

| Characteristics | 3 months | 6 months |
| --- | --- | --- |
| Number of infants | 127 | 126 |
| Number of females | 73 (57.4%) | 71(56.3%) |
| Gestational age at birth ^a^ | 275.5±9.1 | 275.4 ± 9.0 |
| Birth weight ^a^ | 3043±342.6 | 3948.3 ± 340.4 |
| Maternal antimicrobial use at delivery | 69 (55.1%) | 68 (54%) |
| Caesarean section | 27 (21.3%) | 27 (21.4%) |
| Premature rupture of membrane | 19 (15%) | 21 (16.7%) |
| Group B Streptococcus positive status | 26 (20.5%) | 30 (23.8%) |
| Infants with older siblings | 66 (52%) | 66 (52.4%) |
| Exclusive breast feeding | 78 (61.4%) | 77 (61.1%) |
| Age of mothers ^a^ | 31.5±5.1 | 31.5 ± 5.1 |
| Maternal history of allergy | 55 (43.4%) | 53 (42.1%) |
| Neonatal respiratory disorder | 2 (1.6%) | 2 (1.6%) |
| Neonatal jaundice | 4 (3.1%) | 4 (3.2%) |
| RSV infection | 2 (1.6%) | 2 (1.6%) |
| Maternal history of smoking | 9 (7%) | 8 (6.3%) |
| Maternal history of hyperthyroidism | 2 (1.6%) | 2 (1.6%) |
| Maternal history of diabetes Mellitus | 2 (1.6%) | 2 (1.6%) |

^a^ Gestational age at birth, birth weight, and maternal age are shown as the mean and standard deviation. All other data are shown as the number of subjects and a percentage.

RSV: respiratory syncytial virus

Supplementary Table S2 online. Influence of background factors of the mother and child at 3 and 6 months on occupancies of the top five bacterial genera in the infant’s intestinal microbiome.

| 3 months old Variances | | *Bifidobacterium* | | | | | | | *Bacteroides* | | | | | | | *Escherichia* | | | | | | | | *Streptococcus* | | | | | | | *Ruminococcus* | | | | |
| --- | --- | --- | --- | --- | --- | --- | --- | --- | --- | --- | --- | --- | --- | --- | --- | --- | --- | --- | --- | --- | --- | --- | --- | --- | --- | --- | --- | --- | --- | --- | --- | --- | --- | --- | --- |
|  | | Odds Ratio | | 95% CI | | | | | Odds Ratio | | 95% CI | | | | | Odds Ratio | | 95% CI | | | | | | Odds Ratio | | 95% CI | | | | | Odds Ratio | | 95% CI | | |
| Gestational days ^a^ | 1 | 1.01 | | 0.96 | | 1.07 | |  | 0.91 | | 0.85 | | 0.97 | | * | 0.99. | | 0.94 | | 1.05 | |  | | 1.05 | | 0.99 | | 1.11 | |  | 1.01. | | 0.97 | 1.01 |  |
|  | 2 | 0.98 | | 0.93 | | 1.04 | |  | 0.90 | | 0.84 | | 0.96 | | * | 1.01 | | 0.95 | | 1.06 | |  | | 1.06 | | 1.00 | | 1.12 | |  | NA | | NA | NA |  |
|  | 3 | 0.98 | | 0.93 | | 1.04 | |  | 0.95 | | 0.90 | | 1.01 | |  | 1.02 | | 0.96 | | 1.07 | |  | | 1.03 | | 0.98 | | 1.10 | |  | 0.99 | | 0.94 | 1.05 |  |
| Birth weight ^a^ | 1 | 1.00 | | 0.998 | | 1.001 | |  | 0.999. | | 0.997. | | 1.00 | |  | 1.00 | | 0.998. | | 1.001 | |  | | 1.000 | | 0.999. | | 1.001 | |  | 1.000 | | 0.999 | 1.002 |  |
|  | 2 | 0.999 | | 0.998 | | 1.001 | |  | 0.999 | | 0.997 | | 1.00 | |  | 1.001 | | 0.999 | | 1.002 | |  | | 1.000 | | 0.999 | | 1.002 | |  | NA | | NA | NA |  |
|  | 3 | 0.999 | | 0.998 | | 1.001 | |  | 1.00 | | 0.998 | | 1.001 | |  | 1.000 | | 0.999 | | 1.001 | |  | | 1.000 | | 0.998 | | 1.001 | |  | 0.998 | | 0.997 | 1.000 |  |
| Age of mothers ^a^ | 1 | 0.99 | | 0.90 | | 1.09 | |  | 1.06 | | 0.96 | | 1.17 | |  | 1.04 | | 0.94 | | 1.15 | |  | | 0.97 | | 0.88 | | 1.07 | |  | 0.95 | | 0.87 | 1.03 |  |
|  | 2 | 0.96 | | 0.87 | | 1.06 | |  | 1.03 | | 0.94 | | 1.14 | |  | 0.99 | | 0.89 | | 1.10 | |  | | 0.97 | | 0.88 | | 1.07 | |  | NA | | NA | NA |  |
|  | 3 | 0.94 | | 0.85 | | 1.04 | |  | 0.998 | | 0.91 | | 1.10 | |  | 1.03 | | 0.93 | | 1.13 | |  | | 1.02 | | 0.92 | | 1.12 | |  | 1.00 | | 0.90 | 1.11 |  |
| Male | | 0.64 | | 0.21 | | 1.97 | |  | 1.41 | | 0.62 | | 3.21 | |  | 1.43 | | 0.59 | | 3.47 | |  | | 0.49 | | 0.23 | | 1.04 | |  | 1.00 | | 0.46 | 2.19 |  |
| Vaginal delivery | | 1.19 | | 0.25 | | 5.70 | |  | 0.14 | | 0.03 | | 0.64 | | * | 0.32 | | 0.08 | | 1.21 | |  | | 1.42 | | 0.41 | | 4.84 | |  | 2.14 | | 0.61 | 7.46 |  |
| Infants without siblings | | 3.73 | | 1.11 | | 12.58 | | * | 0.94 | | 0.41 | | 2.18 | |  | 2.24 | | 0.84 | | 5.97 | |  | | 1.30 | | 0.59 | | 2.88 | |  | 0.84 | | 0.37 | 1.91 |  |
| Non-AED | | 0.30 | | 0.09 | | 1.00 | | * | 0.69 | | 0.29 | | 1.65 | |  | 0.66 | | 0.24 | | 1.84 | |  | | 1.39 | | 0.59 | | 3.27 | |  | 1.02 | | 0.42 | 2.46 |  |
| Mothers without allergy | | 1.77 | | 0.57 | | 5.50 | |  | 0.78 | | 0.34 | | 1.79 | |  | 0.75 | | 0.30 | | 1.83 | |  | | 1.04 | | 0.47 | | 2.27 | |  | 1.46 | | 0.66 | 3.23 |  |
| Exclusively breast fed | | 2.17 | | 0.67 | | 7.04 | |  | 1.85 | | 0.77 | | 4.44 | |  | 1.01 | | 0.40 | | 2.51 | |  | | 0.91 | | 0.41 | | 2.01 | |  | 2.52 | | 1.13 | 5.62 |  |
| 6 months old variances | | *Bifidobacterium* | | | | | | *Bacteroides* | | | | | | | *Streptococcus* | | | | | | | | *Enterobacteriaceae; other* | | | | | | | *Ruminococcus* | | | | | |
|  | | Odds Ratio | 95% CI | | | | | Odds Ratio | | 95% CI | | | | | Odds Ratio | | 95% CI | | | | | | Odds Ratio | | 95% CI | | | | | Odds Ratio | | 95% CI | | | |
| Gestational days ^a^ | 1 | 0.99 | 0.97 | | 1.02 | |  | 0.999 | | 0.998 | | 1.000 | |  | 0.99 | | 0.97 | | 1.02 | |  | | 0.97 | | 0.92 | | 1.02 | |  | 0.99 | | 0.97 | | 1.02 |  |
|  | 2 | 1.00 | 0.97 | | 1.03 | |  | 1.000 | | 0.998 | | 1.002 | |  | 1.00 | | 0.97 | | 1.03 | |  | | 1.01 | | 0.95 | | 1.07 | |  | NA | | NA | | NA |  |
|  | 3 | 1.00 | 0.97 | | 1.03 | |  | 0.999 | | 0.998 | | 1.001 | |  | 1.00 | | 0.97 | | 1.03 | |  | | 0.98 | | 0.93 | | 1.03 | |  | 1.00 | | 0.96 | | 1.03 |  |
| Birth weight ^a^ | 1 | 1.000 | 0.999 | | 1.001 | |  | 1.000 | | 0.999 | | 1.001 | |  | 1.000 | | 0.999 | | 1.001 | |  | | 0.999 | | 0.998 | | 1.000 | |  | 1.000 | | 0.999. | | 1.003 |  |
|  | 2 | 1.000 | 0.999 | | 1.001 | |  | 1.000 | | 0.999 | | 1.001 | |  | 1.000 | | 0.999 | | 1.001 | |  | | 1.000 | | 0.999 | | 1.001 | |  | NA | | NA | | NA |  |
|  | 3 | 1.000 | 0.999 | | 1.001 | |  | 1.000 | | 0.999 | | 1.001 | |  | 1.000 | | 0.999 | | 1.001 | |  | | 0.999 | | 0.998 | | 1.001 | |  | 1.000 | | 0.999 | | 1.001 |  |
| Age of mothers ^a^ | 1 | 1.06 | 0.97 | | 1.16 | |  | 0.96 | | 0.92 | | 1.01 | |  | 0.97. | | 0.90 | | 1.07 | |  | | 1.00 | | 0.91 | | 1.09 | |  | 0.93 | | 0.86 | | 1.01 |  |
|  | 2 | 1.03 | 0.94 | | 1.12 | |  | 1.02 | | 0.95 | | 1.10 | |  | 0.95 | | 0.87 | | 1.04 | |  | | 0.98 | | 0.90 | | 1.10 | |  | NA | | NA | | NA |  |
|  | 3 | 1.04 | 0.95 | | 1.14 | |  | 0.99 | | 0.93 | | 1.04 | |  | 1.01 | | 0.92 | | 1.11 | |  | | 0.94 | | 0.86 | | 1.03 | |  | 0.90 | | 0.81 | | 1.00 |  |
| Male | | 1.86 | 0.87 | | 3.98 | |  | 0.52 | | 0.24 | | 1.14 | |  | 1.38 | | 0.64 | | 2.99 | |  | | 0.64 | | 0.30 | | 1.40 | |  | 1.62 | | 0.72 | | 3.65 |  |
| Vaginal delivery | | 1.83 | 0.52 | | 6.44 | |  | 2.19 | | 0.61 | | 7.83 | |  | 0.26 | | 0.07 | | 0.91 | | * | | 0.44 | | 0.12 | | 1.54 | |  | 0.30 | | 0.08 | | 1.14 |  |
| Infants without siblings | | 0.70 | 0.31 | | 1.54 | |  | 0.55 | | 0.24 | | 1.24 | |  | 0.40 | | 0.18 | | 0.88 | | * | | 1.72 | | 0.77 | | 3.87 | |  | 1.47 | | 0.63 | | 3.44 |  |
| Non-AED | | 1.01 | 0.43 | | 2.37 | |  | 1.29 | | 0.55 | | 3.05 | |  | 1.53 | | 0.65 | | 3.63 | |  | | 1.39 | | 0.59 | | 3.27 | |  | 1.64 | | 0.65 | | 4.14 |  |
| Mothers without allergy | | 1.48 | 0.68 | | 3.22 | |  | 0.65 | | 0.29 | | 1.43 | |  | 1.01 | | 0.46 | | 2.23 | |  | | 2.67 | | 1.20 | | 5.94 | | * | 0.51 | | 0.22 | | 1.17 |  |
| Exclusively  breast fed | | 1.70 | 0.77 | | 3.74 | |  | 0.37 | | 0.16 | | 0.84 | | * | 1.47 | | 0.66 | | 3.28 | |  | | 1.14 | | 0.51 | | 2.52 | |  | 0.29 | | 0.12 | | 0.66 | ** |

The bacterial genera are shown from left to right in the order of higher occupancy (mean). For the continuous variables (^a^ gestational age at birth, birth weight, and maternal age), occupancy was classified from 1–4 in ascending order from low occupancy with the occupancy of each bacterial species rounded to four decimal places. For the high occupancy group (group 4), a multinomial logistic regression analysis was performed as the category standard and the occupancy was classified into two groups based on the median for each genus for the logistic regression analysis for categorical variable. The odds ratio and 95% confidence interval were calculated by logistic regression analysis, and the multinomial regression analysis. The significance level was set at 5%. *p <0.05, **p <0.01, ***p <0.001.

NA: not available

Supplementary Table S3 online. Background factors of the mother and infant were compared between groups with and without antibiotic exposure at delivery (AED vs non-AED).

| AED vs  Non-AED | 1 month (N = 130) | |  |  | 3 months (N = 127) | |  |  | 6 months (N = 126) | |  |
| --- | --- | --- | --- | --- | --- | --- | --- | --- | --- | --- | --- |
|  | AED | non-AED | P |  | AED | non-AED | P |  | AED | non-AED | P |
| Number of infants | 74 | 56 |  |  | 69 | 58 |  |  | 68 | 58 |  |
| Number of females | 37 (50) | 33 (59) | 0.31 |  | 36 (52.2) | 36 (62.1) | 0.29 |  | 36 (52.9) | 36 (62.1) | 0.47 |
| Gestational age  at birth | 272.4 (10.1) | 278.8 (6.5) | < 0.001 |  | 272.4 (9.8) | 279.2 (6.2) | < 0.001 |  | 272.5 (9.9) | 279.2 (6.2) | < 0.001 |
| Birth weight | 2989.7 (371.5) | 3104.1 (279.6) | 0.08 |  | 2974.3 (371.1) | 3125.4 (284.2) | 0.01 |  | 2981.2 (369.4) | 3125.4 (284.2) | 0.02 |
| ABPC | 43 (58.1) | 0 | NA |  | 42 (60.9) | 0 | NA |  | 40 (58.8) | 0 | NA |
| CEZ | 31 (41.9) | 0 | NA |  | 27 (39.1) | 0 | NA |  | 28 (41.2) | 0 | NA |
| Infants with older siblings | 44 (59.5) | 24 (42.9) | 0.051 |  | 42 (60.9) | 24 (41.4) | 0.034 |  | 41 (60.3) | 24 (41.4) | 0.07 |
| Exclusive breast feeding | 42 (56.8) | 38 (67.9) | 0.21 |  | 37 (53.6) | 41 (70.7) | 0.067 |  | 37 (54.4) | 41 (70.7) | 0.1 |
| Age of mothers | 32.6 (4.9) | 30.3 (5.2) | 0.01 |  | 32.3 (4.8) | 30.6 (5.2) | 0.045 |  | 32.5 (4.7) | 30.6 (5.2) | 0.02 |

A Mann–Whitney U-test was used for continuous variables (gestational age at delivery, birth weight, and maternal age). A Pearson chi-square test was used for categorical variables (number of girls, infants with older siblings, and exclusive breastfeeding).

Supplementary Table S4 online. Upper row: Comparison of background factors between ampicillin (ABPC)-treated (Vaginal delivery, VD) and cefazolin (CEZ)-treated (caesarean section, CS) groups in infants at each age with antibiotic exposure at delivery (AED).

Lower row: Comparison of background factors of the mother and infant between groups with (AED) and without antibiotic exposure (non-AED) at vaginal delivery (VD) at each age.

| AED group | 1M | |  |  | 3M | |  |  | 6M | |  |
| --- | --- | --- | --- | --- | --- | --- | --- | --- | --- | --- | --- |
|  | ABPC (VD) | CEZ (CS) | P |  | ABPC (VD) | CEZ (CS) | P |  | ABPC (VD) | CEZ (CS) | P |
| Number of infants | 43 | 31 |  |  | 42 | 27 |  |  | 40 | 28 |  |
| Number of females | 20 (46.5) | 17 (54.8) | 0.63 |  | 20 (47.6) | 16 (59.3) | 0.46 |  | 20 (50) | 16 (57.1) | 0.46 |
| Gestational age at birth | 278 (7) | 264.6 (8.6) | < 0.001 |  | 277.1 (6.9) | 265 (9.1) | < 0.001 |  | 277.7 (6.6) | 265 (9.1) | <0.001 |
| Birth weight | 3125.5 (306) | 2801.4 (372) | < 0.001 |  | 3101 (324.5) | 2777.1 (352.6) | < 0.001 |  | 3127.6 (309.2) | 2777.1 (265) | <0.01 |
| Infants with older siblings | 21 (48.8) | 23 (74.2) | 0.03 |  | 21 (50) | 21 (77.8) | 0.03 |  | 19 (47.5) | 21 (75) | 0.03 |
| Exclusive breast feeding | 24 (55.8) | 18 (58.1) | 1 |  | 23 (54.8) | 14 (51.9) | 1 |  | 22 (55%) | 14 (50) | 0.81 |
| Age of mothers | 31.7 (4.8) | 34 (4.5) | 0.03 |  | 31.6 (4.7) | 33.5 (4.7) | 0.09 |  | 31.7 (4.6) | 33.5 (4.7) | 0.12 |

| VD group | 1M | |  |  | 3M | |  |  | 6M | |  |
| --- | --- | --- | --- | --- | --- | --- | --- | --- | --- | --- | --- |
|  | AED | Non-AED | P |  | AED | Non-AED | P |  | AED | Non-AED | P |
| Number of infants | 43 | 56 |  |  | 42 | 58 |  |  | 40 | 58 |  |
| Number of females | 20 (46.5) | 33 (59) | 0.23 |  | 20 (47.6) | 36 (62.1) | 0.16 |  | 20 (50) | 36 (62.1) | 0.31 |
| Gestational age at birth | 278 (7) | 278.8 (6.5) | 0.74 |  | 277.1 (6.9) | 279.2 (6.2) | 0.22 |  | 277.7 (6.6) | 279.2 (6.2) | 0.43 |
| Birth weight | 3125.5 (306) | 3104.1 (279.6) | 0.67 |  | 3101 (324.5) | 3125.4 (284.2) | 0.63 |  | 3127.6 (309.2) | 3125.4 (284.2) | 0.77 |
| Infants with older siblings | 21 (48.8) | 24 (42.9) | 0.54 |  | 21 (50) | 24 (41.4) | 0.42 |  | 19 (47.5) | 24 (41.4) | 0.68 |
| Exclusive breast feeding | 24 (55.8) | 38 (67.9) | 0.29 |  | 23 (54.8) | 41 (70.7) | 0.14 |  | 22 (55%) | 41 (70.7) | 0.21 |
| Age of mothers | 31.7 (4.8) | 30.3 (5.2) | 0.19 |  | 31.6 (4.7) | 30.6 (5.2) | 0.28 |  | 31.7 (4.6) | 30.6 (5.2) | 0.17 |

A Mann–Whitney U-test was used for continuous variables (gestational age at delivery, birth weight, and maternal age). A Pearson chi-square test was used for categorical variables (number of girls, infants with older siblings, and exclusive breastfeeding).
